# Development and validation of an instrument for tracking and analyzing recovery from symptoms associated with COVID-19

**DOI:** 10.1590/S2237-96222025v34e20240220.en

**Published:** 2025-08-04

**Authors:** Isabel Dielle Souza Lima Pio, Deuzilane Muniz Nunes, Josilenne Ferreira Barros, Fabrício Souza Silva

**Affiliations:** 1 Universidade Estadual de Feira de Santana, Programa de Pós-Graduação em Biotecnologia, Feira de Santana, BA, Brazil; 2 Universidade Federal do Vale do São Francisco, Colegiado do Curso de Farmácia, Petrolina, PE, Brazil; 3 Universidade Federal do Vale do São Francisco, Programa de Residência Multiprofissional em Intensivismo, Petrolina, PE, Brazil

**Keywords:** Post-Acute COVID-19 Syndrome, Signs and Symptoms, Test Reproducibility, Surveys and Questionnaires, Validation Study, Síndrome Post Agudo de Covid-19, Signos y Síntomas, Reproducibilidad de los Resultados, Encuestas y Cuestionarios, Estudio de Validación

## Abstract

**Objective:**

To develop and validate the psychometric properties of a screening and recovery instrument for signs and symptoms associated with COVID-19 or post-acute COVID-19 syndrome.

**Methods:**

This study consisted of four stages: literature mapping; development of the instrument; content validation; and assessment of internal consistency and criterion validity for some items. Content validation was conducted via an online panel, using the Likert scale and the Delphi technique for concordance, summarized by the content validity index. Internal consistency was assessed by calculating and analyzing Cronbach’s alpha coefficient. The analysis of concurrent validity was conducted using Spearman’s and intraclass correlation coefficients.

**Results:**

Twenty publications were selected: 17 observational studies and 3 systematic reviews. The tool prototype was sent for content validation with 23 expert judges. After changes, a content validity index of 0.83 was obtained, indicating excellent concordance. The current version of the Screening and Analysis Form for the Recovery Trajectory of Symptoms Potentially Related to Covid-19 was applied to 70 people six weeks after hospital discharge due to severe COVID-19 and showed reasonable internal consistency (Cronbach’s α=0.76). The items best correlated with the scores of the contrasted instruments or domains were dyspnea (Spearman’s ρ=0.93; p-value 0.007) and malaise (intraclass correlation coefficient=-0.73; 95% confidence interval -2.44; 0.30).

**Conclusion:**

The instrument had satisfactory content and correlation validity for the items compared.

## Resumen

**Objetivo:**

Desarrollar y validar las propiedades psicométricas de un instrumento de detección y recuperación de signos y síntomas asociados con covid-19 o síndrome post agudo de covid-19.

**Métodos:**

Este estudio se divide en cuatro etapas: mapeo literario; preparación del instrumento; validación de contenido; y evaluación de la consistencia interna y validez de criterio para algunos ítems. La validación de contenido se realizó mediante un panel online, utilizando la escala Likert y la técnica Delphi para concordancia, resumida en el índice de validez de contenido. La consistencia interna se evaluó mediante el cálculo y análisis del coeficiente alfa de Cronbach. El análisis de validez concurrente se realizó mediante los coeficientes de correlación de Spearman e intraclase.

**Resultados:**

Se seleccionaron 20 publicaciones: 17 estudios observacionales y 3 revisiones sistemáticas. El prototipo de la herramienta fue enviado para validación de contenido con 23 jueces expertos. Luego de los cambios se obtuvo un índice de validez de contenido de 0,83, lo que indica un excelente acuerdo. La versión actual del Formulario de Detección y Análisis de la Trayectoria de Recuperación de Síntomas Potencialmente Relacionados con Covid-19 se aplicó a 70 personas seis semanas después del alta hospitalaria por covid-19 grave y mostró una consistencia interna razonable (α de Cronbach = 0,76). Los ítems que mejor correlacionaron con las puntuaciones de los instrumentos o dominios contrastados fueron disnea (ρ de Spearman=0,93; p-valor 0,007) y malestar (coeficiente de correlación intraclase=-0,73; intervalo de confianza del 95% -2,44; 0,30).

**Conclusión:**

El instrumento presentó validez de contenido y correlación satisfactoria para los ítems comparados.

**Ethical aspects**

This research respected ethical principles, having obtained the following approval data::

Research Ethics Committee: Hospital Agamenon Magalhães

Opinion number: 4.948.225

Approval date: 1/9/2021

Certificate of Submission for Ethical Appraisal: 42078620.8.0000.5197

Informed Consent Form: Obtained from all participants prior to collection of samples.

## Introduction

As the COVID-19 pandemic continues, reports have emerged of people who developed or maintained symptoms related to the disease after the acute phase of the infection (1). Post-acute COVID-19 syndrome or simply long COVID-19 is a concern, given its potential to significantly impact health, productivity, and quality of life.

It was estimated that a sizable portion of individuals who were hospitalized for COVID-19 developed persistent health issued up to 60 days after discharge (2, 3). The frequency of post-acute COVID-19 syndrome was estimated to affect up to 20.0% of patients (4). Although frequent, the characterization of this disease is challenging, given its heterogeneous manifestation and evolution. Potential causes include: acute-phase organ damage, persistent hyperinflammatory state or “cytokine storm,” preserved viral activity by a virus reservoir in the host, inadequate antibody response (5) and invasive and prolonged hospital care (6). Added to these consequences of acute illness from COVID-19 are some individual characteristics, considered potential predictors of long-term COVID-19: age over 50 years; manifestation of the disease in the first week with more than five symptoms; female gender; and pre-existing lung disease (7).

In Brazil, since the beginning of the pandemic until March 2024, more than 38 million cases of COVID-19 have been registered (8). There may be up to 7.5 million Brazilians with long-term COVID-19 who may still require care for the after-effects and recovery of their quality of life (4). There is a need to track and monitor this population using valid instruments and reproducible measures. The objective of this study was to develop and validate psychometric properties of a screening instrument and recovery trajectory for signs and symptoms potentially associated with COVID-19 or post-acute covid-19 syndrome.

## Methods

The methodological approach was conducted in four stages: literature review on COVID-19 and post-acute COVID-19 syndrome, development of the instrument, content validation using the Delphi technique (9), and assessment of reliability via internal consistency and concurrent criterion validity for some criteria.

### Literature review and instrument development

Keywords were chosen from the Health Sciences Descriptors thesaurus of the Virtual Health Library to search the databases: Online Medical Literature Analysis and Retrieval System, Online Scientific Electronic Library, and the Periodicals Portal of the Coordination for the Improvement of Higher Education Personnel of the Ministry of Education. The terms “COVID-19”, “post-acute COVID-19 syndrome”, “long COVID-19” and “persistent symptoms” were used, connected by Boolean operators. Complete peer-reviewed articles, available in Portuguese and English, published between December 2019 and February 2021 were retrieved. Pre-publication articles available on online public servers from Cold Spring Harbor Laboratory, in the areas of biological sciences (https://www.biorxiv.org/) and medical (https://www.medrxiv.org/) were also included.

After selecting and analyzing the literary material, the researchers synthesized the pertinent information, proceeding with conceptual mapping and detection and measurement strategies. Through the iterative process, it was decided by consensus which items would best capture the construct in a concise and objective way, and thus, the development of the prototype of the instrument began.

### Content validation

An online panel was conducted with expert judges, using the Delphi technique to obtain consensus (9). Fifty invitations were sent to higher-level healthcare professionals working in units of the interstate public healthcare network in Pernambuco and Bahia, intentionally selected for their experience with care for COVID-19 and its outcomes. Invitation letters were sent via smartphone instant messaging app and by e-mail. For the next rounds, the form was sent to those experts who had responded in the previous stage.

The judgment was based on three criteria: clarity of language, which assessed the adequacy, intelligibility and exact expression of what was expected to be measured; practical relevance, which observed whether the prototype items concerned the phenomenon of interest; and scope, a criterion that analyzed whether the items addressed all dimensions of the problem (10). The evaluators assessed individually the content of the instrument using a Likert scale with three levels of concordance: 1) disagree; 2) indifferent; and 3) agree. The possibility of suggesting deleting, modifying, or including new items was offered. All suggestions from experts were considered by the team to decide whether to accept them, with justification. The instrument evaluation form was circulated in three rounds between October 2022 and February 2023.

The content validation index was used, as it allows the analysis of each item individually and the instrument as a whole (10). Values greater than 0.70 in the three criteria established satisfactory concordance. Modified questions or those with an index lower than 0.70 were resubmitted in the subsequent round until the value was reached or they were excluded from the instrument.

### Assessment of internal consistency and concurrent validation

### Population and sample

Individuals of any sex (biological classification assigned at birth) and race/skin color, aged between 18-60 years, who had confirmed COVID-19 by diagnostic examination and who were admitted to intensive care units in hospital institutions in two neighboring cities located in Bahia and Pernambuco were recruited. Individuals who were discharged due to recovery within six weeks prior to the start of this study were eligible. People were approached by telephone, informed about the research, and invited to participate in it.

### Procedures and data analysis

Sociodemographic and clinical data were collected from medical records and recorded in a specific electronic form. The instrument was applied in person by a single researcher who requested individuals to report the presence and intensity of symptoms during the acute phase of COVID-19 and at the time of contact. Specific instruments were applied for dyspnea (modified Medical Research Council dyspnea scale) (11), fatigue (fatigue severity scale) (12) and perception of quality of life (World Health Organization quality of life questionnaire, abbreviated version) (13).

The reliability of the instrument was verified using Cronbach’s alpha coefficient for the general scale (all items) and for each of the extracted factors (14). The measurement of this coefficient varies from 0 to 1. Values between 0.70 and 0.90 convey satisfactory internal consistency (15).

Concurrent validity, a type of criterion validity (15), was assessed by the correlation between some items of the newly created instrument (fatigue, dyspnea and malaise) and another validated and freely available instrument (or domain): dyspnea (11) and fatigue scales (12) and the physical domain of the World Health Organization quality of life questionnaire (13). All tools were applied on the same day and were conducted by the same researcher. Spearman’s coefficient was adopted for correlation analyses since the variables did not present normal distribution (according to the Kolmogorov-Smirnov test) and intraclass correlation coefficient. The correlation was considered moderate for values of 0.30-0.59; substantial for 0.60-0.79; and almost complete for values ≥0.80 (16,17).

All analyses were performed using the Statistical Package for the Social Sciences software, version 24.0 (SPSS Inc, Chicago, Illinois, United States). The level of statistical significance was set at p-value<0.05.

## Results

20 original publications were selected: 17 observational studies and 3 systematic reviews (18-20), all published in English, mostly from Europe. Latin American authors produced only 1 work (19) (Supplementary Table 1).

**Table 1:** Absolute and percentage distribution of the 17 signs and symptoms related to acute COVID-19 or post-acute COVID-19 syndrome cited in the selected studies. Brazil, December 2019 - February 2021 (n=20)

Signs and symptoms	n (%)
Fatigue	16 (80.0)
Mental health-related conditions: irritability, sleep disorders, anxiety, depression, post-traumatic stress	11 (55.0)
Cognitive disorders: mental confusion, inattention, memory loss	10 (50.0)
Dyspnea	11 (55.0)
Body aches	7 (35.0)
Cough	7 (35.0)
Anosmia or hyposmia	7 (35.0)
Headache	6 (30.0)
Fever	6 (30.0)
Discomfort	4 (20.0)
Ageusia or dysgeusia	4 (20.0)
Sore throat	3 (15.0)
Nasal congestion	2 (10.0)
Diarrhea	2 (10.0)
Nausea or vomiting	1 (5.0)
Hair loss	1 (5.0)
Testicular pain	1 (5.0)

The objectives of the work focused on suggesting physical or psychosocial consequences of COVID-19. Three of them were about tools specifically aimed at people with COVID-19 (21-23). Two studies had their focus on the convalescent population: one was an instrument for assessing functional capacity after illness and the other for telemonitoring persistent signs and symptoms. (22,23). Data collection techniques ranged from interviews and free narrative (24) to questionnaires with dichotomous answers and numerical scale (23).

As different definitions were found for post-acute COVID-19 syndrome, we decided to use the one established by the UK health authorities: “signs and symptoms that develop during or after an illness consistent with COVID-19, continue for more than four weeks and are not explained by alternative diagnoses” (25). The purpose of the instrument was to detect clinical manifestations in the various organ systems, to circumscribe acute or long COVID-19 without establishing a definitive diagnosis or classifying by severity. This tool should also indicate when these symptoms are reduced or resolved, once applied at two or more separate occasions.

The literature survey revealed a list of 17 signs and symptoms used to develop the prototype of the instrument. Symptoms of fatigue, dyspnea and those related to mental health were cited by more than half of the selected studies (Table 1). The symptoms of COVID-19 defined by the Brazilian Ministry of Health protocols were considered (3). In search of a correspondence with the tools and studies analyzed, we prepared a questionnaire-type instrument, combining two response strategies: i) a question with a binary answer (“yes, no”) to measure the existence of the sign or symptom; and ii) an 11-point scale from 0 to 10 to verify the intensity (where 0 represents “I do not have this issue” and 10, “this symptom is very significant”). This construction was based on the Edmonton Symptom Assessment System instrument, which assesses multiple symptoms of cancer patients undergoing palliative treatment (26).

The semantic adaptation of the instrument was conducted in an iterative process that sought everyday nomenclatures, in addition to adding explanatory texts in parentheses. An example of this was the description of the item “ageusia/dysgeusia,” adapted to “change in taste (change in the taste of food).”

The instrument was divided into two sections. Section A had its focus on acute COVID-19, and participants were asked to answer whether they feel or have felt each symptom and define its maximum intensity. Section B addressed screening for persistent signs and symptoms that could be related to post-acute COVID-19 syndrome. Between the two sections, the question was arranged to verify individual perception prior to the acute illness: “Did you have any of these symptoms immediately before you had COVID-19? Which?”.

The “instructions” section was structured with a methodological description of the application of the tool and a warning text for cases of persistent symptoms that suggest a worsening of the individual’s health status. Two informative paragraphs called “To the researcher/investigator” were added and can be found at each section. The prototype was preliminarily named Screening and Trajectory Form for Symptoms Potentially Associated with COVID-19. This was forwarded to the content validation stage.

Of the 50 invitations sent for content validation, 46.0% (23 judges) responded. The percentages of 60.9% (n=14) and 56.5% (n=13) persisted in the second and third rounds. Among the participants in the first round, there was a predominance of women (65.2%), pharmacists (60.9%), with a median time of professional practice of four (2-15 years) years. The majority (56.0%) were professionals caring for patients with COVID-19 at the time of the study (Table 2). 

**Table 2:** Characterization of volunteers in the content validation stage of the Screening and Analysis Form for the Recovery Trajectory of Symptoms Potentially Related to Covid-19 (RRS-COVID-19). Bahia and Pernambuco, 2023 (n=23)

Descriptors	n (%)
Gender	
Female	15 (65.2)
Male	8 (34.8)
**Professional category**	
Pharmaceutical	14 (60.9)
Nutritionist	1 (4.3)
Nurse	3 (13.0)
Physiotherapist	3 (13.0)
Physician	1 (4.3)
Others	1 (4.3)
Length of professional experience in years (median and interquartile range)	4 (2-15)
**Are you currently working in a COVID-19 unit**?	
Yes	13 (56.0)
No	10 (44.0)

Most items evaluated in the first round (69.2%) presented a content validity index greater than 0.70 in the three criteria assessed and were promptly validated. The proposed strategy to verify the presence of signs and symptoms (with a “yes, no” response) obtained an index of 1.0 for clarity and 0.96 for relevance. All items with a content validity index lower than 0.70 belonged to the signs and symptoms inventory. They were then rewritten and forwarded to the next round.

From the first round of validation, nine suggestions for changes emerged. Three suggestions referred to the term “trajectory” in the instrument’s title (two requested replacement of the term, and one, the inclusion of the word “analysis”); two referred to improvements in the wording of the instructions in section B; two focused on changes in the numerical intensity scale or its text; and two of them suggested the inclusion of new items in the inventory (periorbital pain and glycemic changes). Six suggestions were accepted, because the others did not include a replacement proposal (Supplementary Table 2).

In the second round, there was only one suggestion: changing the item “change in taste”, including the expression “absence of flavor”, which was accepted. The third round took place with six items. At this stage, two items (pain in the testicles and nausea and vomiting) were excluded, due to the persistence of an index lower than 0.70. The version of the instrument with the modifications obtained a total content validity index of 0.83 (Supplementary Table 2).

**Figure 1:**
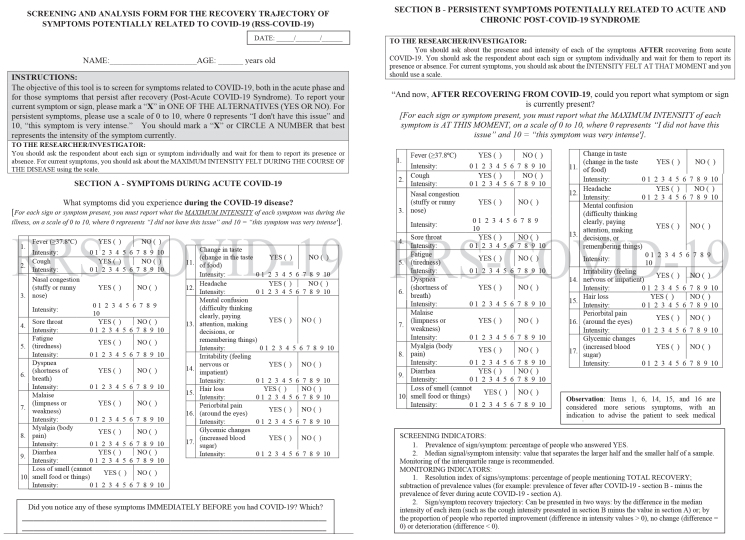
The current version of the Screening and Analysis Form for the Recovery Trajectory of Symptoms Potentially Related to COVID-19 (RRS-COVID-19). Brazil, 2023

The current version of the instrument was titled Screening and Analysis Form for the Recovery Trajectory of Symptoms Potentially Related to Covid-19, abbreviated as RRS-COVID-19 (Figure 1). The format for results presentation was established, summarized through prevalence and intensity medians for each of the signs and symptoms evaluated. Two monitoring indicators were determined, called resolution index and recovery trajectory from the sign or symptom. The resolution index consisted of subtracting prevalence values (for example: prevalence of fever after COVID-19 [section B] minus the prevalence of fever during acute COVID-19 [section A]). 

The recovery trajectory could be presented in two ways: i) by the difference in the median intensity of each item (such as the intensity of the cough presented in section B minus the intensity of the cough in section A); or ii) by the proportion of people who reported improvement (difference in the intensity values>0), no change (difference=0) or deterioration (difference<0). For the exclusive monitoring of post-acute COVID-19 syndrome, this reasoning was used for the measurements collected by section B arranged at separate times.

The instrument was applied to 70 individuals, with an average of 6.23±1.80 weeks after hospital discharge. The sample was homogeneous, composed of a small male majority (54.3%) with a mean age of 53.45±1.78 years and 1.49±1.94 previous comorbidities, with a higher prevalence of arterial hypertension (40.0%) (Supplementary Table 3).

**Table 3:** Monitoring indicators (resolution index and recovery trajectory) of signs and symptoms inventoried by the instrument Screening and Analysis Form for the Recovery Trajectory of Symptoms Potentially Related to Covid-19 (RRS-COVID-19). Bahia and Pernambuco, 2023 (n=70)

Sign/symptom	Resolution index n (%)^a^	Recovery trajectory		
		Recovery	No. change	Deterioration
		n (%)^b^		
Fever	45 (64.0)	3 (4.3)	63 (90.0)	4 (5.7)
Sore throat	26 (37.1)	51 (72.9)	14 (20.0)	5 (7.1)
Change in taste	22 (31.4)	61 (87.1)	6 (8.6)	3 (4.3)
Periorbital pain	17 (24.3)	20 (28.6)	50 (71.4)	0 (0.0)
Myalgia	16 (22.9)	57 (81.4)	6 (8.6)	7 (10.0)
Diarrhea	16 (22.9)	20 (28.6)	50 (71.4)	0 (0.0)
Discomfort	12 (17.1)	51 (72.7)	5 (7.1)	14 (20.0)
Headache	7 (10.0)	51 (72.7)	11 (15.7)	8 (11.4)
Loss of smell	7 (10.0)	52 (74.3)	14 (20.0)	4 (5.7)
Irritability	5 (7.1)	6 (8.6)	64 (91.4)	0 (0.0)
Nasal congestion	2 (2.9)	34 (48.6)	16 (22.9)	20 (28.6)
Fatigue	1 (1.4)	40 (57.1)	10 (14.3)	20 (28.6)
Mental confusion	1 (1.4)	2 (2.9)	66 (94.3)	2 (2.9)
Glycemic changes	1 (1.4)	6 (8.6)	64 (91.4)	0 (0.0)
Dyspnea	1 (1.4)	39 (55.7)	30 (42.86)	1 (1.4)
Cough	0 (0.0)	6 (8.6)	62 (88.6)	2 (2.9)
Hair loss	-10 (-14.3)	1 (1.4)	54 (77.1)	15 (21.4)

^a^Difference between prevalence values found in sections B and A. ^b^Difference between the median intensity of sections B and A of each persistent symptom or sign; the results were distributed into three subgroups: i) recovery (difference in intensity values>0); ii) no change (difference=0); and iii) deterioration (difference<0).

The most prevalent symptoms in acute COVID-19 were malaise, fatigue, changes in taste, fever, and dyspnea, all with a frequency greater than 95.0% in the sample studied (Supplementary Figure 1). The resolution index for cough, fatigue, and dyspnea was below 5.0%, which demonstrated high persistence after hospital discharge. Fever showed a higher resolution index: 64.0%. Hair loss showed a negative index (-14.3%), indicating that 10 people reported this sign after acute COVID-19. Among the persistent symptoms and signs, the change in taste showed the best recovery behavior, as 61 respondents (87.1%) declared a reduction in intensity between the points of evaluation. Fatigue and nasal congestion showed worsening or deterioration for 20 (28.6%) individuals surveyed (Table 3).

The overall Cronbach’s α value, based on the standardized items, was 0.76, which meant reasonable internal consistency (14,15), without major changes with the exclusion of any item. 

The medians of the intensities of the symptoms of fatigue, dyspnea and malaise related to the post-COVID-19 period (section B of the RRS-COVID-19 instrument) were moderately correlated with the scores of the instruments considered as validation criteria, with emphasis on dyspnea with a Spearman’s ρ value of 0.93 (p-value 0.007). The intraclass correlation coefficient was higher for the malaise item (-0.73) and the 95% confidence interval (95%CI) was -2.44; 0.30. This item compares negatively with the physical domain of the quality-of-life questionnaire used, since this tool assesses the individual’s perception of well-being and quality of life (13), a diametrically opposite criterion. Although all responses showed moderate intensity values, the RRS-COVID-19 numbers were slightly higher (Table 4).

**Table 4:** Intensity (median and interquartile range – IQI) and indicators of concurrent validation (Spearman’s ρ and intraclass correlation coefficient) of the fatigue, dyspnea and malaise items Screening and Analysis Form for the Recovery Trajectory of Symptoms Potentially Related to Covid-19 (RRS-COVID-19). Bahia and Pernambuco, 2023 (n=70)

Symptom	RRS-COVID-19 median (IIQ)	Criterion median (IIQ)	Spearman’s ρ (p-value)	Intraclass correlation coefficient (95%CI)^a^)
Fatigue	6.00 (5.00-8.25)	Fatigue Scale (Global Score)^b^ 4.00 (3.00-5.56)	0.50 (<0.001)	0.44 (-0.86; 0.70)
Dyspnea	7.00 (5.00-8.00)	Dyspnea scale (Global Score)^c^ 3.00 (2.00-4.00)	0.93 (0.007)	0.45 (-0.16; 0.78)
Discomfort	6.00 (2.00-8.00)	WHOQoL-BREF^d^ 3.14 (2.43-4.14)	-0.59 (0.006)	-0.73 (-2.44; 0.30)

^a^95% confidence interval; ^b^Fatigue severity scale; ^c^Modified Medical Research Council dyspnea scale; ^d^WHOQoL-BREF: domain I of the World Health Organization quality of life questionnaire, abbreviated version.

## Discussion

This study presented the stages of development and initial evaluation of the instrument, following the methodological recommendations in the literature. It was found that the Screening and Analysis Form for the Recovery Trajectory of Symptoms Potentially Related to Covid-19 (RRS-COVID-19) has content validity, satisfactory reliability, and substantial correlation with the contrasted instruments. 

The totality of publications found in the literature review brought difficulties for the mapping of the construct. This is because the literature review is marked by a high concentration of opinion texts – something to be expected at the beginning of a pandemic of an unknown disease. Cautious selection, considering Evidence-Based Health, led to the construction of a multicriteria tool, which tracks and monitors acute or long-term COVID-19 through the accumulation of 17 manifestations in different organ structures or systems, but in a format that challenges psychometric analysis.

In this study, we recognize that the response rate in content validation was limiting, despite the adoption of careful practices, such as messaging and email reminders. The low adherence of judges could be justified by the remote work during the pandemic period (27). Pharmaceutical training, more common among judges, is not, per se, a problem. This is because the purpose of the instrument was tracking and monitoring, without the intention of conducting a medical diagnosis, like the rapid laboratory tests widely used in community pharmacies during the pandemic.

Another limitation concerns the sample of respondents, all of whom were recovering from severe COVID-19. As the occurrence of post-acute COVID-19 syndrome is already recognized in individuals with mild or even asymptomatic infection (1), it was observed the need to evaluate the performance of RRS-COVID-19 for different population groups.

Cronbach’s α value showed satisfactory reliability, mainly because it is a multidimensional instrument, with independent criterion items. Although it is the most widely used indicator for multiple scales in health, its exclusive use to assess consistency should be analyzed cautiously, given its nature that is influenced by the size and homogeneity of the sample (28).

Concurrent validity was analyzed by correlation with different tools for criterion items, given the absence of gold standard instruments for screening symptoms of acute or long-term COVID-19. Fatigue and dyspnea scales were validated for Brazilians with stable chronic obstructive pulmonary disease (11,12), a population group with characteristics closer to those of people with long COVID-19 than the conditions of cancer patients, the target audience of the Edmonton Symptom Assessment System instrument (26).

There are different definitions for post-acute COVID-19 syndrome that vary mainly in terms of the period of onset of persistent symptoms. To reduce disagreements, the World Health Organization established that long COVID-19 is the continuation or development of new symptoms after 12 weeks of the acute disease, with a minimum duration of two months (27). The heterogeneity of the presentation of COVID-19 and its complications, together with the short post-pandemic time to study recovered cases, is problematic for the development and adaptation of clinical instruments. In Brazil, some researchers are paying attention to this, assessing the viability and discriminatory capacity of the Post-COVID-19 Functional Scale instrument (29) or the epidemiological indicator Disability-Adjusted Life Year, related to the global burden of disease (30). As of the end of this work, no national study evaluating tools for detecting and monitoring symptoms of long COVID-19 was found. Hence the novelty of this study.

In the context of long COVID-19, there is a need to measure more than binary results such as having or not having a certain condition or mortality. For this purpose, a consensus was established that the mixed approach with dichotomous measurement and ordinal polytomous scale would be ideal for considering the full range of manifestations of this condition. These scales can be used both for self-response and in interviews and are suitable for clinical research, obtaining quantitative data with the least amount of stress for the respondent.

Among the signs and symptoms inventoried by the instrument, typical manifestations of acute morbidity were observed, such as fever, myalgia and malaise, but these were reported between 13 and 114 days after recovery (2,27). It is possible that such issues persist due to the consequences of acute COVID-19, its treatment and individual vulnerability resulting from previous illnesses (3).

Neural involvement by viral infection causes acute complaints and can lead to psychosocial problems, also covered in the instrument, through the comprehensive items “mental confusion” and “irritability.” The relevance of this lies in the fact that illness is a state of vulnerability to cognitive and psychological changes that impact quality of life. The COVID-19 pandemic scenario has led to a worsening of the mental health status of the entire society. Clinical-epidemiological research into the mental health burden is necessary to readjust policies and services (30).

These are the first steps towards creating a robust and reliable instrument for Brazilian reality, with the potential to be used in routine healthcare work. Future initiatives will evaluate other parameters of reliability and validity of this version, as well as its updates and adaptations.

In view of the urgent need to adequately characterize COVID-19, whether acute or long-term, the RRS-COVID-19 instrument is innovative in that it makes it possible to produce comparable, consistent, and reproducible information to support strategies in the field of science and healthcare.

## Data Availability

The data related to the article can be accessed through the link, as well as the database related to the literature review, the table of proposed changes in the content validation process, the other characteristics of the volunteers from the internal consistency and concurrent validation assessment stage, and the current version of the instrument: https://drive.google.com/drive/folders/12lTNISpKCF6tQSX-ulw_qD0HINEAfLxp?usp=sharing

## Supplementary material

Supplementary Table 1, Supplementary Table 2, Supplementary Table 3 and Supplementary Figure 1.
