# Supplementary material for: Development and validation of an instrument for tracking and analyzing recovery from symptoms associated with COVID-19
Source: Epidemiol Serv Saude. 2025 Aug 4;34:e20240220. doi: 10.1590/S2237-96222025v34e20240220.en (PMC12334159; doi:10.1590/S2237-96222025v34e20240220.en)
Supplement: Supplementary file 1 [file 2237-9622-ress-34-e20240220-supl01-pt.pdf]

Material suplementar

Tabela suplementar 1. Características gerais dos estudos selecionados para o mapeamento de sinais e sintomas relacionados à covid-19 aguda ou pós-aguda. Brasil, 2022 (n=20)

N	Autor principal/ país; Periódico, ano	Metodologia /amostra	Instrumentos utilizados	Principais resultados
1.	CARFI, A./Itália; JAMA, 2020(2).	Transversal associado a um serviço de acompanhamento.<br>Cento e quarenta e três pacientes que receberam alta por cura da covid-19 aguda sem detecção de SARS-CoV-2[a] em aproximadamente 60 dias após os primeiros sintomas.	Questionário padronizado específico para o rastreio de sintomas.<br>Escala analógica visual genérica para qualidade de vida da EuroQol Research Foundation.	Apenas 12,6% dos pacientes estavam completamente recu-perados, enquanto 32,0% e 55,0% apresentavam um ou dois sintomas e três ou mais. Os principais sintomas foram fadiga, dispneia, dor torácica e articular. Piora da qualidade de vida também foi bastante relatada.
2.	DOYKOV, I./Reino Unido; F1000Research, 2021. (31)	Coorte com abordagem proteômica de marcadores inflamatórios.<br>Amostras séricas de 10 pacientes assintomáticos ou moderadamente doentes, coletadas após 40-60 dias da infecção. Grupo controle de 10 indivíduos com exames negativos para SARS-COV 2[a].	Não se aplica.	Significativa alteração na contagem de proteínas associadas a estresse mitocondrial: a peroxirredoxina 3 se mostrou elevada, enquando que carbamoil fosfato sintase apresentou redução. Contagens elevadas das proteínas: N-Myc downstream regulated gene 1; com repetição de hélice tripla de colágeno e cistatina C. Observou-se diminuição da progranulina sérica. Isso pode indicar que vias inflamatórias e bioquímicas podem permanecer alteradas mesmo após o estabelecimento da cura.
3.	FERRUCCI, R./Itália; Brain Sciences, 2021. (32)	Transversal.	Pergunta sobre anosmia/disgeusia durante e/ou após internação (resposta dicotômica ("Sim/Não"); Montreal Cognitive Assessment,	Observou-se déficit na velocidade de processamento ou redução na memória verbal em 42,1% e 26,3% dos indivíduos. Houve perdas em ambos os domínios em 21,0%

		Trinta e oito pacientes internados por covid-19 em unidade de cuidados intermediários recrutados de quatro a cinco meses após alta hospitalar.	Brief Repeatable Battery of Neuropsychological Tests; Beck's Depression Inventory-II; Subjective Scale of Damage questionnaire.	da amostra. A SARS-CoV2[a] e redução da PAO_2[b] apresentaram correlação positiva com a ocorrência dessas sequelas.
4.	GUEDJ, E./França; European Journal of Nuclear Medicine and Molecular Imaging, 2021.(21)	Caso-controle<br>Grupo de casos: 35 sujeitos com queixas após três semanas de cura da covid-19; Grupo controle: 44 sujeitos saudáveis.	National Early Warning Score, versão adaptada para pacientes com covid-19.	Pacientes com sintomas persistentes (dispneia, dor torácica e muscular, cefaleia, insônia e distúrbios cognitivos e de memória) apresentaram imagens de PET-CT[c] com hipometabolismo do marcador 18-FDG[d] em várias regiões do cérebro. Essa condição demonstrou ser um diferencial entre os dois grupos, com 100,0% de classificação correta entre os indivíduos com e sem queixas.
5.	HALPIN, S. J./Reino Unido; Journal of Medical Virology, 2020.(23)	Transversal associado a serviço de acompanhamento.<br>Cem indivíduos que receberam alta por cura da covid-19 aguda entre quatro e oito semanas antes do início do estudo. Destes, 68 foram tratados em enfermarias e 32 estiveram em unidade de terapia intensiva (UTI).	Covid-19 Yorkshire Rehabilitation Screening.<br>Escala de autoavaliação do estado de saúde, com cinco dimensões e três níveis de resposta da EuroQol Research Foundation.	Fadiga foi o sintoma persistente mais comum relatado por 72,0% dos pacientes que haviam sido internados na UTI e 60,3% do grupo tratado em enfermaria. Dispneia também foi bastante frequente (65,6% no grupo UTI e 42,6% no grupo enfermaria), seguido por sofrimento psíquico pós-traumático (mais comum em jovens). Sintomas relacionados à comunicação, voz, deglutição e tosse persistente foram mais comuns no grupo UTI. Observou-se queda significativa na qualidade de vida.
6.	HOLTMANN, N./Alemanha; Fertility and Sterility, 2020.(33)	Coorte<br>Trinta e quatro homens foram recrutados para coleta de sangue e sêmen: 18 homens recuperados (8-54 dias após a ausência de sintomas); 14 indivíduos controle e dois	Não informado.	Dezessete dos 18 participantes recuperados descreveram sintomas, principalmente febre, tosse, cefaleia, dor muscular, dispneia e fadiga. Dois participantes apresentaram leve anosmia e ageusia. Um participante relatou dor testicular. Indivíduos com infecção moderada ativa

		pacientes com covid-19 (infecção ativa moderada).		apresentaram significativo impacto na qualidade do sêmen, diferentemente dos pacientes curados e controle.
7.	KINGSTONE, T./Reino Unido; British Journal of General Practitioners Open, 2020.(24)	Transversal com metodologia qualitativa. Vinte e quatro indivíduos que reportavam sintomas persistentes, recrutados via mídias sociais.	Roteiro de entrevista semiestruturada para contato por telefone ou videoconferência.	Os relatos descrevem a dificuldade de compreender e manejar os sintomas persistentes (dor torácica, fadiga, distúrbios cognitivos/de memória) e a procura por diferentes fontes de ajuda (apoio de pares, suporte *online*, terapias complementares, dietas especiais e suplementos. Outros achados foram o sentimento de desamparo, medo sobre a recuperação e a necessidade de acesso a profissionais de saúde habilitados para o cuidado com as sequelas.
8.	LADDS, E./Reino Unido; BioMed Central health services research, 2020.(34)	Transversal com metodologia qualitativa. Cento e catorze indivíduos com sintomas há mais de três semanas após a cura da covid-19 aguda foram recrutados nas redes sociais. Realizaram-se 55 entrevistas individuais e oito grupos focais com 59 participantes.	Não informado.	Demonstraram-se principalmente a confusão sobre o diagnóstico e as dificuldades de se estabelecer um prognóstico para síndrome pós-covid-19 aguda. Sintomas como fadiga e confusão mental mostraram-se frequentes. Aventaram-se também sentimento de perda, estigma e dificuldade de acesso aos serviços de saúde.
9.	LÓPEZ-LÉON S./México; SSRN, 2021.(19)	Revisão sistemática com meta-análise de 15 estudos que reportavam sintomas e sinais persistentes há mais de duas semanas em grupos de pacientes que tiveram covid-19.	Não se aplica.	Realizaram-se 21 metanálises com 47.910 pacientes, detectando que 80,0% dos pacientes infectados com SARS-CoV-2[a] desenvolveram um ou mais dos 55 sintomas persistentes, com duração entre 14 e 110 dias após a infecção viral. Os sintomas mais comuns foram fadiga, dor de cabeça, distúrbio de atenção, queda de cabelo e dispneia.

10.	LUDVIGSSON, J./Suécia; Acta Paediatrica, 2020.(18)	Relato de casos e revisão sistemática de 19 trabalhos publicados entre 1º de janeiro e 18 de março de 2020.	Não se aplica.	Cinco crianças apresentaram fadiga, dispneia, palpitações cardíacas ou dor no peito, e quatro tinham cefaleia, dificuldades de concentração, fraqueza muscular, tonturas e dores de garganta. Na revisão, nenhum estudo apresentou dados sobre síndrome pós-covid-19 aguda em crianças.
11.	MACHADO, F./Holanda; Health and Quality of Life Outcomes, 2021.(22)	Transversal de validação de construto. Mil novecentos e trinta e nove indivíduos com sintomas persistentes há três meses após a doença aguda foram convidados mediante redes sociais e registros hospitalares.	Questionário *online* composto por: Post-Covid-19 Functional Status Scale; Utrecht Symptom Diary; Assessment System; escala de autoavaliação do estado de saúde, com cinco dimensões e três níveis de resposta da EuroQol Research Foundation; Work Productivity and Activity Impairment.	A maioria (85,0%) apresentou limitações leves, moderadas ou graves. Os sintomas persistentes com maior intensidade (fadiga, fraqueza muscular e distúrbios do sono) se associaram a piores escores dos instrumentos contrastados. A associação mais forte ocorreu entre a presença dos sintomas e o domínio "atividades habituais" da escala de autoavaliação do estado de saúde, da EuroQol Research Foundation.
12.	MANDAL, S./Reino Unido; Thorax Published Online First, 2020. (35)	Coorte. Trezentos e oitenta e quatro pacientes que receberam alta hospitalar por cura foram acompanhados pessoal ou remotamente por 54 dias.	Instrumento pró-forma de registros de dados clínicos. Escala de dispneia do Medical Researcher Center.	Reportaram-se fadiga (69,0% da amostra), dispneia (53,0%) e tosse (34,0%), concentração elevada de dímero D e proteína C reativa; 38,0% das radiografias de tórax permaneceram anormais, com 9,0% em franca deterioração.
13.	MAZZA, M. G./Itália; Brain, Behavior, and Immunity, 2020.(36)	Transversal associado a coorte. Grupo de 402 pacientes recuperados da covid-19 acompanhado por aproximadamente 30 dias após alta ou avaliação no unidade de emergência hospitalar.	Impact of Events Scale-Revised; Post-Traumatic Stress Disorder Checklist for DSM-5[e]; Zung Self-Rating Depression Scale; 13-item Beck's Depression Inventory; State-Trait Anxiety Inventory form Y; Medical Outcomes Study Sleep Scale; Women's Health	O acompanhamento revelou a presença dos seguintes sintomas: ansiedade (42,0%); insônia (40,0%), depressão (31%), estresse pós-traumático (28,0%) e transtorno obsessivo-compulsivo (20,0%). As mulheres sofreram mais com ansiedade e depressão, e pacientes com diagnóstico psiquiátrico prévio mostraram escores maiores para testes psicopatológicos.

			Initiative Insomnia Rating Scale; e Obsessive-Compulsive Inventory.	
14.	SOLLINI, M./Itália; European Journal of Nuclear Medicine and Molecular Imaging, 2020.(37)	Caso-controle.<br>Grupo caso: 10 indivíduos com sintomas persistentes por pelo menos 30 dias.<br>Grupo controle: 10 pacientes oncológicos.	Não informado.	Os indivíduos do grupo caso relataram febre, dispneia, tosse, fadiga e anosmia/ageusia.<br>A captação de 18-FDG[d] via PET-CT[e] na medula óssea e o escore vascular não diferiram entre os grupos.
15.	SYKES, D. L./Reino Unido; Lung, 2021.(38)	Coorte.<br>Cento e trinta e quatro pacientes que receberam alta hospitalar foram acompanhados por aproximadamente 113 dias.	Instrumento pró-forma de registros de dados clínicos de acompanhamento.<br>Escala de dispneia do Medical Research Center.<br>Escala de autoavaliação do estado de saúde, com cinco dimensões e três níveis de resposta da EuroQol Research Foundation.	Do grupo, 86,0% relataram pelo menos um sintoma, independentemente da gravidade da covid-19 aguda. Dispneia, mialgia, ansiedade, fadiga, redução do humor e distúrbios do sono foram os sintomas mais comuns. O sexo feminino e IMC[f] elevado associaram-se à fadiga e mialgia.
16.	TABACOF, L./Estados Unidos, MedRxiv, 2020.(39)	Transversal.<br>Cento e cinquenta e seis indivíduos que foram atendidos em um ambulatório para covid-19 aguda participaram da pesquisa 351 dias após a infecção.	Questionário *online* com os seguintes instrumentos: Fatigue Severity Scale; Fatigue Visual Analog Scale; escala de dispneia do Medical Research Center; sistema de medição de qualidade de vida em distúrbios neurológicos da equipe HealthMeasures da Northwestern University; escala de autoavaliação do estado de saúde, com cinco dimensões e três níveis de resposta da EuroQol Research Foundation; Generalized Anxiety Disorder Scale; Patient Health Questionnaire-	Os sintomas persistentes mais relatados foram fadiga, confusão mental e dor de cabeça. Os desencadeadores mais comuns de exacerbação dos sintomas foram esforço físico, estresse e desidratação.

			2; World Health Organization Disability Assessment Schedule; e dois instrumentos criados pela equipe dessa pesquisa: Completion of Regular Moderate and Vigorous Intensity Physical Activity e Pré–and Post-Covid-19 Employment Status.	
17.	TENFORDE, M.W./Estados Unidos; Morbidity and Mortality Weekly Report, 2020. (40)	Transversal.<br>Duzentos e setenta e quatro indivíduos positivos para infecção por SAR-CoV-2[a] foram entrevistados entre 14 e 21 dias após o diagnóstico.	Não informado. Contudo, as entrevistas de triagem e sintomas basearam-se no informativo da CDC[g] (2020) intitulado: Coronavirus disease 2019 (covid-19). Symptoms of Coronavirus.	Entre os entrevistados, 35,0% não haviam retornado ao seu estado de saúde anterior à infecção. Os sintomas mais relatados foram fadiga, tosse e cefaleia. Febre e calafrios foram os sintomas que resolveram mais rapidamente. Tosse e fadiga persistiram por mais tempo.
18.	VENTURELLI, S./Itália; Epidemiology and Infection, 2021.(41)	Transversal associado a uma coorte.<br>Setecentos e sessenta e sete pacientes recuperados da covid-19 foram acompanhados por 81 dias após alta hospitalar	Impact of Events Scale - Revised; Hospital Anxiety and Depression Scale e; Resilience Scale for Adults.	Do total do grupo, 51,4% apresentavam sequelas, como fadiga e dispneia ao esforço, e 30,5% apresentavam adoecimento psicológico. A função pulmonar estava prejudicada em 19,0% da amostra, e 17,0% tinham valores de dímero D duas vezes acima do limite para diagnóstico de embolia pulmonar, com dois casos de trombose pulmonar silenciosa.
19.	WILLI, S./Suíça;<br>Travel Medicine and Infectious Disease, 2021.(20)	Revisão sistemática.<br>Trinta e uma publicações de estudos incluíram estudos observacionais transversais, de coorte prospectivos e retrospectivos e relatos de casos de adultos menores de anos.	Não se aplica.	A persistência das sequelas variou de 14 a 120 dias. Relataram-se fadiga, dispneia, diminuição da qualidade de vida, disfunção pulmonar com resultados anormais em tomografia, sintomas neurológicos, peri/perimio/miocardite, aumento da

				incidência de diagnósticos psiquiátricos, recuperação incompleta da anosmia e ageusia.
20.	YAN, C. H./Estados Unidos; Otolaryngology - Head and Neck Surgery, 2020(42).	Transversal.<br>Trezentos e dezesseis indivíduos considerados curados da covid-19 há 9,4 dias.	Questionário aplicado por telefone. Os indivíduos avaliaram seu olfato em uma escala de 10 pontos (0, anosmia completa; 10, olfato normal) em três momentos diferentes: antes de adoecer por covid-19; no momento do início dos sintomas ou testagem; e na data atual da pesquisa.	Anosmia foi relatada por 50,0% das pessoas no decurso da covid-19 aguda, mas 78,0% destas relataram persistência desse sintoma, no momento do estudo.

Notas: [a]*Severe acute respiratory syndrome coronavirus* 2; [b]Pressão parcial de oxigênio no sangue arterial; [c]*Positron emission tomography – computed tomography*; [d]18-Fluorodesoxiglicose; [e]Diagnostic and Statistical Manual of Mental Disorders-5 version; [f]Índice de massa corporal; [g]Center for Disease Control and Prevention.

Referências da tabela suplementar 1

1. Carfi A, Bernabei R, Landi F, Gemelli Against COVID-19 Post-Acute Care Study Group. Persistent symptoms in patients after acute COVID-19. JAMA. 2020;324(6):603-5.

2. Doykov I, Hällqvist J, Gilmour KC, Grandjean L, Mills K, Heywood WE. "The long tail of covid-19" - The detection of a prolonged inflammatory response after a SARS-CoV-2 infection in asymptomatic and mildly affected patients. F1000Res. 2020;9:1349.

3. Ferrucci R, Dini M, Groppo E, Rosci C, Reitano MR, Bai F, et al. Long-lasting cognitive abnormalities after COVID-19. Brain Sci. 2021;11(2):235.

4. Guedj E, Campion JY, Dudouet P, Kaphan E, Bregeon F, Tissot-Dupont H, et al. 18F-FDG brain PET hypometabolism in patients with long COVID. Eur J Nucl Med Mol Imaging. 2021; 48(9):2823-33.

5. Halpin SJ, McIvor C, Whyatt G, Adams A, Harvey O, McLean L, et al. Postdischarge symptoms and rehabilitation needs in survivors of covid-19 infection: A cross-sectional evaluation. J Med Virol. 2021; 93(2):1013-22.

6. Holtmann N, Edimiris P, Andree M, Doehmen C, Baston-Buest D, Adams O, et al. Assessment of SARS-CoV-2 in human sêmen – a cohort study. Fertil Steril. 2020;114(2):233-8.

7. Kingstone T, Taylor AK, O'Donnell CA, Atherton H, Blane DN, Chew-Graham CA. Finding the 'right' GP: a qualitative study of the experiences of people with long-COVID. BJGP Open. 2020;4(5):bjgpopen20X101143.

8. Ladds E, Rushforth A, Wieringa S, Taylor S, Rayner C, Husain L, et al. Persistent symptoms after covid-19: Qualitative study of 114 "long Covid" patients and draft quality criteria for services. BMC Health Serv Res. 2020;20:1-13.

9. Lopez-Leon S, Wegman-Ostrosky T, Perelman C, Sepulveda R, Rebolledo PA, Cuapio A, et al. More than 50 Long-Term Effects of Covid-19: a systematic review and meta-analysis. Sci Rep. 2021;11(1):16144.

10. Ludvigsson JF. Case report and systematic review suggest that children may experience similar long-term effects to adults after clinical COVID-19. Acta Paediatr. 2021;110(3):914-21.

11. Machado FVC, Meys R, Delbressine JM, Vaes AW, Goërtz YMJ, Herck M, et al. Construct validity of the post-COVID-19 functional status scale in adult subjects with COVID-19. Health Qual Life Outcomes. 2021;19(1):40.

12. Mandal S, Barnett J, Brill SE, Brown JS, Denneny EK, Hare SS, et al. 'Long-covid': a cross-sectional study of persisting symptoms, biomarker and imaging abnormalities following hospitalisation for covid-19. Thorax. 2021;76(4):396-8.

13. Mazza MG, Lorenzo R, Conte C, Poletti S, Vai B, Bollettini I, et al. Anxiety and depression in COVID-19 survivors: Role of inflammatory and clinical predictors. Brain Behav Immun. 2020; 89:594-600.

14. Sollini M, Ciccarelli M, Cecconi M, Aghemo A, Morelli P, Gelardi F, et al. Vasculitis changes in COVID-19 survivors with persistent symptoms: an [18F]FDG-PET/CT study. Eur J Nucl Med Mol Imaging. 2021;48(5):1460-6.

15. Sykes DL, Holdsworth L, Jawad N, Gunasekera P, Morice AH, Crooks MG. Post-COVID-19 Symptom Burden: What is long-COVID and how should we manage it? Lung. 2021;199(2):113-9.

16. Tabacof L, Tosto-Mancuso J, Wood J, Cortes M, Kontorovich A, McCarthy D, et al. Post-acute COVID-19 syndrome negatively impacts health and wellbeing despite less severe acute infection. medRxiv [Internet]. 2020 [cited 2021 Mar 21]. Available from: http://medrxiv.org/content/early/2020/11/06/2020.11.04.20226126.abstract

17. Tenforde MW, Kim SS, Lindsell CJ, Rose EB, Shapiro NI, Files DC, et al. Symptom duration and risk factors for delayed return to usual health among outpatients with COVID-19 in a multistate health care systems network - United States, March-June 2020. MMWR Morb Mortal Wkly Rep. 2020;69(30):993-8.

18. Venturelli S, Benatti SV, Casati M, Binda F, Zuglian G, Imeri G, et al. Surviving COVID-19 in Bergamo Province: A post-Acute outpatient re-evaluation. Epidemiol Infect. 2021;149:e-32.

19. Willi S, Lüthold R, Hunt A, Hanggi NV, Sejdiu D, Scaff C, et al. COVID-19 sequelae in adults aged less than 50 years: A systematic review. Travel Med Infect Dis. 2021;40:101995.

20. Yan CH, Prajapati DP, Ritter ML, DeConde AS. Persistent smell loss following undetectable SARS-CoV-2. Otolaryngol Head Neck Surg. 2020;163(5):923-5.

Tabela suplementar 2. Índice da validade de conteúdo (IVC) do instrumento Formulário de Rastreio e Análise da Trajetória de Recuperação de Sintomas Potencialmente Relacionados à Covid-19 (RRS-COVID-19), conforme os critérios de clareza, pertinência e abrangência. Brasil, 2023

Primeira rodada (n=23)				
Itens do instrumento	Critérios (IVC)			IVC total
	Clareza de linguagem	Pertinência	Abrangência	
Título (questão 1)	0,83	0,83	0,78	0,81
Instruções/descrição do instrumento (questão 2)	0,87	0,87	0,83	0,86
Instrução ao pesquisador/investigador - seção A (questão 3)	0,74	0,96	0,7	0,80
Pergunta dirigida aos sintomas da covid-19 aguda – seção A (questão 4)[a]	0,70	0,74	0,78	0,74
Inventário de sintomas (questões 5-21)[b]	0,70	0,42	0,51	0,54
Pergunta de resposta binária “sim,não” para os sintomas (questão 22)	1	0,96	0,82	0,93
Escala numérica de intensidade dos sintomas (questão 23)	0,89	0,79	0,83	0,83
Percepção individual quanto aos sintomas antes da infecção (questão 24)[c]	0,78	0,70	0,86	0,78
Instrução ao pesquisador/investigador – seção B (questão 25)	0,78	0,70	0,74	0,74
Perguntas dirigida aos sintomas persistentes (questão 26)[d]	0,74	0,74	0,7	0,73
Segunda rodada (n=14)				
Itens do instrumento	Critérios (IVC)			IVC total
	Clareza de linguagem	Pertinência	Abrangência	
Título (questão 1)	0,92	0,95	0,92	0,95
Dor de garganta (questão 8)	0,86	0,92	0,81	0,86
Enjoo ou vômito (questão 13)	0,32	0,77	0,53	0,54
Diarreia (questão 14)	0,68	0,61	0,79	0,69
Cefaleia (questão 17)	0,89	0,76	0,92	0,87
Confusão mental – dificuldade de pensar com clareza, prestar atenção, tomar decisão ou lembrar das coisas (questão 18)	0,54	0,74	0,79	0,69

Irritabilidade – sensação de nervoso ou impaciência (questão 19)	0,56	0,73	0,68	0,66
Perda de cabelo (questão 20)	0,84	0,90	0,95	0,90
Dor no testículo (questão 21)	0,20	0,17	0,01	0,13
Instrução ao pesquisador/investigador – seção B (questão 25)	0,87	0,95	0,85	0,89
Dor periorbital – próximo aos olhos (questão 27)	0,77	0,69	0,85	0,77
Alterações glicêmicas – aumento do açúcar no sangue (questão 28)	0,69	0,77	0,92	0,79
Terceira rodada (n=13)				
Itens do instrumento	Critérios (IVC)			IVC total
	Clareza de linguagem	Pertinência	Abrangência	
Enjoo ou vômito (questão 13)	0,85	0,22	0,53	0,53
Diarreia (questão 14)	0,95	0,72	0,81	0,83
Alteração no paladar – mudança do gosto da comida ou ausência de sabor (questão 16)	0,72	0,87	1	0,86
Confusão mental – dificuldade de pensar com clareza, prestar atenção, tomar decisão ou lembrar das coisas (questão 18)	0,88	0,85	0,74	0,82
Irritabilidade – sensação de nervoso ou impaciência (questão 19)	0,69	0,81	0,88	0,79
Dor no testículo (questão 21)	0,70	0,15	0,01	0,29
IVC da versão final				0,83

Notas: [a]Referente à questão: “Quais os sintomas você sentiu *durante a fase aguda* (você deve relatar qual foi a intensidade máxima de cada sintoma durante a doença.)?”; [b]Referente ao somatório dos 17 sintomas e sinais potencialmente relacionados à covid-19; [c]Referente à questão: “Você percebia algum desses sintomas IMEDIATAMENTE ANTES de ter covid-19? Qual?”; [d]Referente à questão: “E agora, após a cura da covid-19, você deve informar qual sintoma ou sinal está presente no momento? Para o sintoma persistente, você deve fazer uso de uma escala de 0 a 10, onde 0 representa ‘Não tenho esse problema’ e 10, ‘este sintoma é muito significativo’”.

Tabela suplementar 3. Caracterização dos voluntários na etapa de avaliação da consistência interna e validação concorrente do Formulário de Rastreio e Análise da Trajetória de Recuperação de Sintomas Potencialmente Relacionados à Covid-19 (RRS-COVID-19). Bahia e Pernambuco, 2023 (n=70)

Descritores	n (%)
Sexo	
Masculino	38 (54,3)
Feminino	32 (46,7)
Idade em anos (média±desvio-padrão)	53,27±14,78
IMC[a] kg/cm^2 [mediana (IIQ[b])]	27,86 (20,18-3,98)
Número de comorbidades prévias (média±desvio-padrão)	1,49±1,94
Morbidades diagnosticadas (CID-11)[c, d]	
Hipertensão arterial (BA00.Z)	28 (40,0)
Obesidade (5C1Z)	15 (21,4)
Diabetes (5A14)	13 (18,6)
Dislipidemia (5C80.2)	5 (7,1)
Asma (CA22)	4 (5,7)
Comorbidades associadas à hipertensão arterial[c] (CID-11)[d]	
Diabetes (5A14)	10 (35,7)
Obesidade (5C1Z)	5 (17,9)
Tempo de internamento na UTI[e] em dias (média±desvio-padrão)	9,90±9,61
Uso de ventilação invasiva [N (%)]	62 (88,6)
Fármacos utilizados (média±desvio-padrão)	8,03±5,72
Pacientes que usaram antibióticos [n (%)]	46 (65,7)

Notas: [a]Índice de massa corporal; [b]Intervalo interquartílico; [c]Citadas por mais de 5,0% dos indivíduos; [d]Classificação Internacional de Doenças e Problemas Relacionados à Saúde, 11ª edição; [e]Unidade de tratamento intensivo.

Figura suplementar 1. Prevalência dos sinais e sintomas inventariados pelo Formulário de Rastreio e Análise da Trajetória de Recuperação de Sintomas Potencialmente Relacionados à Covid-19 (RRS-COVID-19). Bahia e Pernambuco, 2023 (n=70)

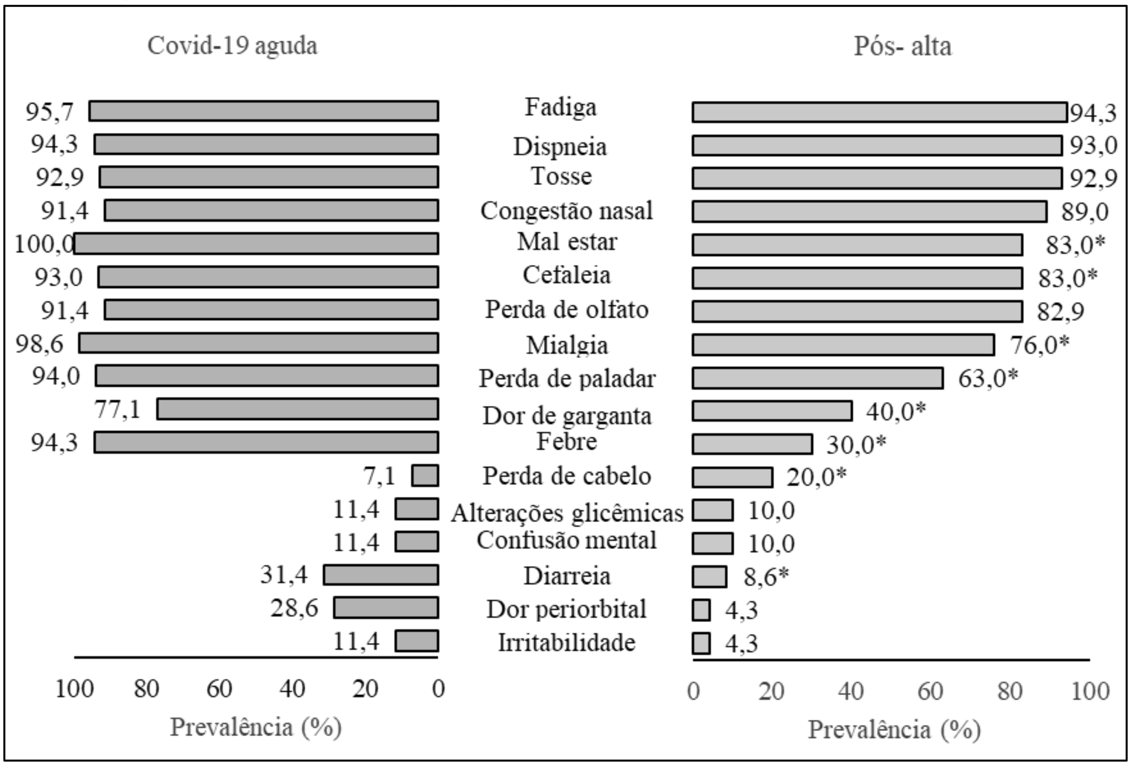

Nota: * Significativo para α=0,05; teste Q de Cochran para amostras relacionadas.
