# Supplementary material for: Development and validation of an instrument for tracking and analyzing recovery from symptoms associated with COVID-19
Source: Epidemiol Serv Saude. 2025 Aug 4;34:e20240220. doi: 10.1590/S2237-96222025v34e20240220.en (PMC12334159; doi:10.1590/S2237-96222025v34e20240220.en)
Supplement: Translation

# Translation: Elaboração e validação de instrumento para rastreio e análise da recuperação de sintomas associados à covid-19

**DOI:** 10.1590/S2237-96222025v34e20240220.pt

**Authors:** Isabel Dielle Souza Lima Pio, Deuzilane Muniz Nunes, Josilenne Ferreira Barros, Fabrício Souza Silva

**Affiliations:** 1 Universidade Estadual de Feira de Santana, Programa de Pós-Graduação em Biotecnologia, Feira de Santana, BA, Brasil; 2 Universidade Federal do Vale do São Francisco, Colegiado do Curso de Farmácia, Petrolina, PE, Brasil; 3 Universidade Federal do Vale do São Francisco, Programa de Residência Multiprofissional em Intensivismo, Petrolina, PE, Brasill

**Keywords:** Síndrome de Covid-19 Pós-Aguda, Sinais e Sintomas, Reprodutibilidade dos Testes, Inquéritos e Questionários, Estudo de Validação

## Abstract

**Objetivo:**

Elaborar e validar propriedades psicométricas de instrumento de rastreio e recuperação de sinais e sintomas associados à covid-19 ou à síndrome de covid-19 pós-aguda.

**Métodos:**

Trata-se de estudo dividido em quatro etapas: mapeamento literário; elaboração do instrumento; validação de conteúdo; e avaliação da consistência interna e validade de critério para alguns itens. Realizou-se a validação do conteúdo via painel online, utilizando a escala Likert e a técnica de Delphi para concordância, sumarizada pelo índice de validade de conteúdo. A consistência interna foi avaliada por meio de cálculo e análise do coeficiente alfa de Cronbach. A análise da validade concorrente ocorreu mediante os coeficientes de correlação de Spearman e intraclasse.

**Resultados:**

Selecionaram-se 20 publicações: 17 estudos observacionais e 3 revisões sistemáticas. O protótipo da ferramenta foi encaminhado para validação de conteúdo com 23 juízes especialistas. Após alterações, obteve-se índice de validade de conteúdo igual a 0,83, denotando ótima concordância. A versão atual do Formulário de Rastreio e Análise da Trajetória de Recuperação de Sintomas Potencialmente Relacionados à Covid-19 foi aplicada em 70 pessoas após seis semanas da alta hospitalar por covid-19 grave e apresentou razoável consistência interna (α de Cronbach=0,76). Os itens mais bem correlacionados com os escores dos instrumentos ou domínios contrastados foram dispneia (ρ de Spearman=0,93; p-valor 0,007) e mal-estar (coeficiente de correlação intraclasse=-0,73; intervalo de confiança de 95% -2,44; 0,30).

**Conclusão:**

O instrumento possuiu satisfatória validade de conteúdo e correlação para os itens comparados.

**Aspectos éticos**

A presente pesquisa respeitou os princípios éticos, obtendo os seguintes dados de aprovação: :

Comitê de ética em pesquisa: Hospital Agamenon Magalhães

Número do parecer: 4.948.225

Data de aprovação: 1/9/2021

Certificado de apresentação de apreciação ética: 42078620.8.0000.5197

Registro do consentimento livre e esclarecido: Obtido de todos os participantes antes da coleta.

## Introdução

Com a continuidade da pandemia de covid-19, surgiram apontamentos de pessoas que desenvolveram ou mantiveram sintomas relacionados à doença após a fase aguda da infecção (1). A síndrome de covid-19 pós-aguda ou simplesmente covid-19 longa é uma preocupação, dado seu potencial de impactar significativamente a saúde, produtividade e qualidade de vida.

Estimou-se que parcela significativa dos indivíduos que foram internados por covid-19 desenvolveram problemas persistentes até 60 dias após alta (2,3). Considerou-se que a frequência da síndrome de covid-19 pós-aguda poderia chegar até 20,0% (4). Embora frequente, a caracterização dessa doença é desafiadora, dada sua heterogênea manifestação e evolução. As potenciais causas são: danos a órgãos na fase aguda, estado hiperinflamatório ou “tempestade de citocinas” persistente, atividade viral preservada por reservatório de vírus no hospedeiro, resposta inadequada de anticorpos (5) e assistência hospitalar invasiva e prolongada (6). Somam-se a essas consequências do adoecimento agudo por covid-19 algumas características individuais, consideradas potenciais preditores para a covid-19 longa: idade maior que 50 anos; manifestação, na primeira semana da doença, de mais de cinco sintomas; sexo feminino; e preexistência de doenças pulmonares (7).

No Brasil, desde o início da pandemia até março de 2024, registraram-se mais de 38 milhões de casos de covid-19 (8). É possível a existência de até 7,5 milhões de brasileiros com covid-19 longa que podem ainda requerer cuidados para as sequelas e recuperação da qualidade de vida (4). Necessita-se rastrear e monitorar essa população, com a utilização de instrumentos válidos e medidas reprodutíveis. Neste estudo, objetivou-se elaborar e validar propriedades psicométricas de instrumento de rastreio e trajetória de recuperação de sinais e sintomas potencialmente associados à covid-19 ou à síndrome de covid-19 pós-aguda.

## Métodos

O percurso metodológico foi conduzido em quatro etapas: revisão literária sobre covid-19 e síndrome da covid-19 pós-aguda, elaboração do instrumento, validação de conteúdo, utilizando a técnica Delphi (9), e avaliação da confiabilidade via consistência interna e validade de critério do tipo concorrente para alguns critérios.

### Revisão da literatura e elaboração do instrumento

Foram escolhidas palavras-chave do tesauro Descritores em Ciências da Saúde, da Biblioteca Virtual em Saúde para busca nas bases de dados: Medical Literature Analysis and Retrieval System Online, Scientific Electronic Library Online e Portal de Periódicos da Coordenação de Aperfeiçoamento de Pessoal de Nível Superior do Ministério da Educação. Utilizaram-se os termos “covid-19”, “síndrome da covid-19 pós-aguda”, “covid-19 longa” e “sintomas persistentes”, conectados por operadores booleanos. Foram resgatados artigos completos revisados por pares, disponíveis nos idiomas português e inglês, publicados entre dezembro de 2019 e fevereiro de 2021. Foram incluídos também artigos em pré-publicação, disponíveis nos servidores públicos online do Cold Spring Harbor Laboratory, das áreas das ciências biológicas (https://www.biorxiv.org/) e médicas (https://www.medrxiv.org/).

Após seleção e análise do material literário, os pesquisadores sintetizaram as informações pertinentes, procedendo o mapeamento conceitual e as estratégias de detecção e mensuração. Por meio do processo iterativo, decidiram-se por consenso quais itens capturariam melhor o construto de forma concisa e objetiva, elaborando o protótipo do instrumento.

### Validação de conteúdo

Realizou-se painel online com juízes especialistas, utilizando a técnica de Delphi para obtenção do consenso (9). Foram enviados 50 convites para profissionais de saúde de nível superior trabalhadores em unidades da rede pública interestadual de saúde de Pernambuco e da Bahia, selecionados intencionalmente pela sua experiência com cuidados em covid-19 e suas sequelas. As cartas-convites foram enviadas via aplicativo de celular smartphone de mensagem instantânea e por e-mail. Para as rodadas seguintes, enviou-se o formulário para aqueles especialistas que haviam respondidos na etapa anterior.

O julgamento se deu para três critérios: clareza de linguagem, o qual avaliou a adequação, inteligibilidade e expressão exata do que se esperava medir; pertinência prática, que observou se os itens do protótipo concernem ao fenômeno de interesse; e abrangência, critério que analisou se os itens abordavam todas as dimensões do problema (10). Os avaliadores julgaram individualmente o conteúdo do instrumento por meio de escala Likert de três níveis de concordância: 1) discordo; 2) indiferente; e 3) concordo. Foi ofertada a possibilidade de sugestão para excluir, modificar ou incluir novos itens. Todas as sugestões dos especialistas foram consideradas pela equipe para a decisão de acatar ou não mediante justificativa. O formulário de avaliação do instrumento circulou em três rodadas entre outubro de 2022 e fevereiro de 2023.

Utilizou-se o índice de validação de conteúdo, uma vez que este permite analisar cada item individualmente e o instrumento como um todo (10). Valores maiores que 0,70 nos três critérios estabeleceram concordância satisfatória. As questões modificadas ou com índice menor que 0,70 foram reenviadas na rodada subsequente até se atingir o valor ou foram excluídas do instrumento.

### Avaliação da consistência interna e validação concorrente

### População e amostra

Foram recrutados indivíduos de qualquer sexo (classificação biológica atribuída no nascimento) e raça/cor da pele, com idade entre 18-60 anos, que apresentaram covid-19 confirmada por exame diagnóstico e que foram internados em unidades de tratamento intensivo em instituições hospitalares de duas cidades vizinhas localizadas na Bahia e em Pernambuco. Foram elegíveis indivíduos que receberam alta por cura em até seis semanas antes do início deste estudo. As pessoas foram abordadas via contato telefônico, informadas e convidadas a participar da pesquisa.

### Procedimentos e análise dos dados

Os dados sociodemográficos e clínicos foram coletados em prontuários médicos e registrados em formulário eletrônico específico. A aplicação do instrumento foi realizada presencialmente por única pesquisadora que solicitou o relato dos indivíduos sobre a presença e intensidade dos sintomas durante a fase aguda de covid-19 e no momento do contato. Foram aplicados instrumentos específicos para dispneia (escala modificada de dispneia do Medical Research Council) (11), fadiga (escala de gravidade de fadiga) (12) e percepção de qualidade de vida (questionário de qualidade de vida da Organização Mundial da Saúde, versão abreviada) (13).

A confiabilidade do instrumento foi verificada pelo coeficiente alfa de Cronbach para a escala geral (todos os itens) e para cada um dos fatores extraídos (14). A medida desse coeficiente varia de 0 a 1. Valores entre 0,70 e 0,90 estimam satisfatória consistência interna (15).

A validade concorrente, um tipo de validade de critério (15), foi avaliada pela correlação entre alguns itens do instrumento recém-criado (fadiga, dispneia e mal-estar) e de outro instrumento (ou domínio) validado e disponível gratuitamente: escalas de dispneia (11) e fadiga (12) e o domínio físico do questionário de qualidade de vida da Organização Mundial da Saúde (13). A aplicação de todas as ferramentas ocorreu no mesmo dia e foi feita pela mesma investigadora. Adotou-se o coeficiente de Spearman para as análises de correlação, visto que as variáveis não apresentaram distribuição normal (pelo teste de Kolmogorov-Smirnov) e coeficiente de correlação intraclasse. Considerou-se a correlação moderada para valores de 0,^30^-0,59; substancial para 0,60-0,79; e quase completa para valores ≥0,80 (16,17).

Todas as análises foram realizadas usando o programa Statistical Package for the Social Sciences 24.0 (SPSS Inc, Chicago, Illinois, Estados Unidos). O nível de significância estatística foi estabelecido para p-valor<0,05.

## Resultados

Foram selecionadas 20 publicações originais: 17 estudos observacionais e 3 revisões sistemáticas (18-20), todos publicados em inglês, com origem majoritariamente europeia. Apenas 1 trabalho foi produzido por autores latino-americanos (19) (Tabela suplementar 1).

Os objetivos dos trabalhos se concentraram em aventar consequências físicas ou psicossociais da covid-19. Três deles versavam sobre ferramentas dirigidas especificamente às pessoas com covid-19 (21-23). Dois trabalhos se referiam ao público convalescente: um foi instrumento de avaliação da capacidade funcional pós-doença e outro, de telemonitoramento de sinais e sintomas persistentes (22,23). As técnicas de coleta de dados variaram de entrevista com narrativa livre (24) a questionários com resposta dicotômica e escala numérica (23).

Como foram encontradas diferentes definições para síndrome de covid-19 pós-aguda, adotou-se aquela estabelecida pelas autoridades sanitárias do Reino Unido: “sinais e sintomas desenvolvidos durante ou após uma doença consistente com covid-19 e que continuam por mais de quatro semanas, não sendo explicados por diagnósticos alternativos” (25). Determinou-se o propósito do instrumento de detectar manifestações clínicas nos diversos sistemas orgânicos, a fim de circunscrever covid-19 aguda ou longa sem estabelecer diagnóstico definitivo ou classificar por gravidade. Essa ferramenta deve indicar também quando ocorre a redução ou resolução desses sintomas, uma vez aplicada em dois ou mais momentos distintos.

O levantamento literário retornou a lista de 17 sinais e sintomas utilizados para a elaboração do protótipo do instrumento. Os sintomas fadiga, dispneia e aqueles relacionados à saúde mental foram citados por mais da metade dos estudos selecionados (Tabela 1). Consideraram-se os sintomas da covid-19 definidos pelos protocolos do Ministério da Saúde (3). Buscando a correspondência com as ferramentas e os estudos analisados, elaborou-se o instrumento do tipo questionário, com combinação de duas estratégias de respostas: i) uma pergunta com resposta binária (“sim, não”) para medir a existência do sinal ou sintoma; e ii) uma escala de 11 pontos de 0 a 10 para verificar a intensidade (em que 0 representa “não tenho este problema” e 10, “este sintoma é muito significativo”). Essa construção baseou-se no instrumento Edmonton Symptom Assessment System, que avalia múltiplos sintomas de pacientes oncológicos em tratamento paliativo (26).

**Tabela 1:** Distribuição absoluta e percentual dos 17 sinais e sintomas relacionados à covid-19 aguda ou síndrome da covid-19 pós-aguda citados nos estudos selecionados. Brasil, dezembro de 2019-fevereiro de 2021 (n=20)

Sinais e sintomas	n (%)
Fadiga	16 (80,0)
Condições relacionadas à saúde mental: irritabilidade, distúrbios do sono, ansiedade, depressão, estresse pós-traumático	11 (55,0)
Distúrbios cognitivos: confusão mental, desatenção, perda de memória	10 (50,0)
Dispneia	11 (55,0)
Dores no corpo	7 (35,0)
Tosse	7 (35,0)
Anosmia ou hiposmia	7 (35,0)
Cefaleia	6 (30,0)
Febre	6 (30,0)
Mal-estar	4 (20,0)
Ageusia ou disgeusia	4 (20,0)
Dor de garganta	3 (15,0)
Congestão nasal	2 (10,0)
Diarreia	2 (10,0)
Náusea ou vômito	1 (5,0)
Perda de cabelo	1 (5,0)
Dor testicular	1 (5,0)

A adaptação semântica do instrumento foi feita em processo iterativo que buscou nomenclaturas cotidianas, além da adição de textos explicativos entre parênteses. Um exemplo disso foi a descrição do item “ageusia/disgeusia”, adaptado para “alteração no paladar (mudança do gosto da comida)”.

O instrumento foi dividido em duas seções. A seção A referiu-se à covid-19 aguda, na qual os participantes foram solicitados a responder se sentem ou sentiram cada sintoma e definir a intensidade máxima. A seção B abordou o rastreio de sinais e sintomas persistentes que poderiam estar relacionados com a síndrome da covid-19 pós-aguda. Entre as duas seções, foi disposta a questão para verificar a percepção individual previamente à doença aguda: “Você percebia algum desses sintomas imediatamente antes de ter covid-19? Qual?”.

Estruturou-se a seção “instruções” com descrição metodológica da aplicação da ferramenta e um texto de alerta para casos de persistência de sintomas que sugerem agravamento do estado de saúde do indivíduo. Foram acrescentados dois parágrafos informativos denominados “ao pesquisador/investigador”, presentes nas áreas iniciais das seções. O protótipo foi denominado preliminarmente como Formulário de Rastreio e Trajetória de Sintomas Potencialmente Associados à Covid-19. Este foi encaminhado para a etapa de validação de conteúdo.

Dos 50 convites enviados para a realização de validação de conteúdo, obteve-se o retorno de 46,0% (23 juízes). Persistiram nas segunda e terceira rodadas o percentual de 60,9% (n=14) e 56,5% (n=13). Entre os participantes da primeira rodada, houve o predomínio de mulheres (65,2%), farmacêuticas (60,9%), com mediana de tempo de exercício profissional de quatro (2-15 anos) anos. A maioria (56,0%) atuava no atendimento a pessoas com covid-19 na época do estudo (Tabela 2). 

**Tabela 2:** Caracterização dos voluntários da etapa de validação de conteúdo do Formulário de Rastreio e Análise da Trajetória de Recuperação de Sintomas Potencialmente Relacionados à Covid-19 (RRS-COVID-19). Bahia e Pernambuco, 2023 (n=23)

Descritores	n (%)
Sexo	
Feminino	15 (65,2)
Masculino	8 (34,8)
**Categoria profissional**	
Farmacêutica	14 (60,9)
Nutricionista	1 (4,3)
Enfermeira	3 (13,0)
Fisioterapeuta	3 (13,0)
Médica	1 (4,3)
Outras	1 (4,3)
Tempo de atuação profissional em anos (mediana e intervalo interquartílico)	4 (2-15)
**Trabalha atualmente em unidade covid**-19?	
Sim	13 (56,0)
Não	10 (44,0)

A maioria dos itens avaliados na primeira rodada (69,2%) apresentou índice de validade de conteúdo maior que 0,70 nos três critérios julgados e foram prontamente validados. A estratégia proposta para verificar a presença dos sinais e sintomas (com resposta do tipo “sim, não”) obteve índice de 1,0 para clareza e 0,96 para pertinência. Todos os itens com índice de validade de conteúdo menor que 0,70 pertenciam ao inventário de sinais e sintomas. Foram, então, reescritos e encaminhados para rodada seguinte.

Da primeira rodada de validação, advieram nove sugestões de alteração. Três sugestões se referiram ao termo “trajetória” do título do instrumento (duas solicitavam substituição do termo, e uma, a inclusão da palavra “análise”); duas, à melhoria na redação das instruções da seção B; duas, à alteração da escala numérica de intensidade ou do seu texto; e duas sugeriam inclusão de novos itens ao inventário (dor periorbital e alterações glicêmicas). Foram acatadas seis sugestões, porque as demais não trouxeram proposição de substituição (Tabela suplementar 2).

Na segunda rodada, houve apenas uma sugestão: a alteração do item “alteração no paladar”, incluindo a expressão “ausência de sabor”, o que foi acatado. A terceira rodada ocorreu com seis itens. Nessa etapa, dois itens (dor nos testículos e enjoo e vômito) foram excluídos, devido à manutenção de índice menor que 0,70. A versão do instrumento com as modificações obteve o índice de validade de conteúdo total de 0,83 (Tabela suplementar 2).

A versão atual do instrumento foi intitulada Formulário de Rastreio e Análise da Trajetória de Recuperação de Sintomas Potencialmente Relacionados à Covid-19, cuja abreviatura é RRS-COVID-19 (Figura 1). Estabeleceu-se o formato de apresentação dos resultados, sumarizada por meio de prevalência e medianas de intensidade para cada um dos sinais e sintomas inventariados. Determinaram-se dois indicadores de monitoramento, denominados índice de resolução e trajetória de recuperação do sinal ou sintoma. O índice de resolução consistiu na subtração dos valores de prevalência (por exemplo: prevalência de febre após covid-19 [seção B] menos a prevalência de febre durante a covid-19 aguda [seção A]). 

**Figura 1:** Versão atual do Formulário de Rastreio e Análise da Trajetória de Recuperação de Sintomas Potencialmente Relacionados à Covid-19 (RRS-COVID-19). Brasil, 2023

A trajetória de recuperação pôde ser apresentada de duas maneiras: i) pela diferença das medianas de intensidade de cada item (como intensidade da tosse apresentada na seção B menos a intensidade da tosse da seção A); ou ii) pela proporção de pessoas que referiram melhora (diferença dos valores de intensidade>0), nenhuma mudança (diferença=0) ou deterioração (diferença<0). Para o monitoramento exclusivo da síndrome de covid-19 pós-aguda, utilizou-se esse raciocínio para as medidas coletadas pela seção B dispostas em diferentes momentos.

O instrumento foi aplicado em 70 indivíduos, com a média de 6,23±1,80 semanas após a alta hospitalar. A amostra foi homogênea, composta pela pequena maioria masculina (54,3%) com idade média de 53,45±1,78 anos e 1,49±1,94 comorbidade prévia, com maior prevalência de hipertensão arterial (40,0%) (Tabela suplementar 3).

Os sintomas mais prevalentes na covid-19 aguda foram mal-estar, fadiga, alteração no paladar, febre e dispneia, todos com frequência maior que 95,0% na amostra estudada (Figura suplementar 1). O índice de resolução para tosse, fadiga e dispneia foi abaixo de 5,0%, o que demonstrou elevada persistência após a alta hospitalar. Febre apresentou maior índice de resolução: 64,0%. Perda de cabelo mostrou índice negativo (-14,3%), indicando que 10 pessoas referiram esse sinal após a covid-19 aguda. Entre os sintomas e sinais persistentes, a alteração no paladar apresentou melhor comportamento de recuperação, já que 61 respondentes (87,1%) declararam redução da intensidade entre os momentos avaliados. Fadiga e congestão nasal mostraram piora ou deterioração para 20 (28,6%) indivíduos pesquisados (Tabela 3).

**Tabela 3:** Indicadores de monitoramento (índice de resolução e trajetória de recuperação) dos sinais e sintomas inventariados pelo instrumento Formulário de Rastreio e Análise da Trajetória de Recuperação de Sintomas Potencialmente Relacionados à Covid-19 (RRS-COVID-19). Bahia e Pernambuco, 2023 (n=70)

Sinal/sintoma	Índice de resolução n (%)^a^	Trajetória de recuperação		
		Recuperação	Sem mudança	Deterioração
		n (%)^b^		
Febre	45 (64,0)	3 (4,3)	63 (90,0)	4 (5,7)
Dor de garganta	26 (37,1)	51 (72,9)	14 (20,0)	5 (7,1)
Alteração no paladar	22 (31,4)	61 (87,1)	6 (8,6)	3 (4,3)
Dor periorbital	17 (24,3)	20 (28,6)	50 (71,4)	0 (0,0)
Mialgia	16 (22,9)	57 (81,4)	6 (8,6)	7 (10,0)
Diarreia	16 (22,9)	20 (28,6)	50 (71,4)	0 (0,0)
Mal-estar	12 (17,1)	51 (72,7)	5 (7,1)	14 (20,0)
Cefaleia	7 (10,0)	51 (72,7)	11 (15,7)	8 (11,4)
Perda de olfato	7 (10,0)	52 (74,3)	14 (20,0)	4 (5,7)
Irritabilidade	5 (7,1)	6 (8,6)	64 (91,4)	0 (0,0)
Congestão nasal	2 (2,9)	34 (48,6)	16 (22,9)	20 (28,6)
Fadiga	1 (1,4)	40 (57,1)	10 (14,3)	20 (28,6)
Confusão mental	1 (1,4)	2 (2,9)	66 (94,3)	2 (2,9)
Alterações glicêmicas	1 (1,4)	6 (8,6)	64 (91,4)	0 (0,0)
Dispneia	1 (1,4)	39 (55,7)	30 (42,86)	1 (1,4)
Tosse	0 (0,0)	6 (8,6)	62 (88,6)	2 (2,9)
Perda de cabelo	-10 (-14,3)	1 (1,4)	54 (77,1)	15 (21,4)

^a^Diferença entre os valores de prevalência encontrados nas seções B e A. ^b^ Diferença entre as medianas de intensidade das seções B e A de cada sintoma ou sinal persistente; os resultados foram distribuídos em três subgrupos: i) recuperação (diferença dos valores de intensidade>0); ii) sem mudança (diferença=0); e iii) deterioração (diferença<0).

O valor do α de Cronbach global, com base nos itens padronizados, foi 0,76, o que significou razoável consistência interna (14,15), sem alterações importantes com a exclusão de qualquer item. 

As medianas das intensidades dos sintomas fadiga, dispneia e mal-estar relacionados ao momento pós-covid-19 (seção B do instrumento RRS-COVID-19) foram moderadamente correlacionadas com os escores dos instrumentos considerados critérios de validação, com destaque para dispneia com valor de ρ de Spearman de 0,93 (p-valor 0,007). O coeficiente de correlação intraclasse foi maior para o item mal-estar (-0,73) e o IC95% -2,44; 0,30.Esse item se compara negativamente com o domínio físico do questionário de qualidade de vida usado, visto que essa ferramenta avalia a percepção do indivíduo sobre o bem-estar e a qualidade de vida (13), critério diametralmente oposto. Apesar de todas as respostas mostrarem valores moderados de intensidade, os números do RRS-COVID-19 foram ligeiramente maiores (Tabela 4).

**Tabela 4:** Intensidade (mediana e intervalo interquartílico – IIQ) e indicadores da validação concorrente (ρ de Spearman e coeficiente de correlação intraclasse) dos itens fadiga, dispneia e mal-estar do Formulário de Rastreio e Análise da Trajetória de Recuperação de Sintomas Potencialmente Relacionados à Covid-19 (RRS-COVID-19). Bahia e Pernambuco, 2023 (n=70)

Sintoma	RRS-COVID-19 mediana (IIQ)	Critério mediana (IIQ)	**ρ** de Spearman (p-valor)	Coeficiente de correlação intraclasse (IC95%^a^)
Fadiga	6,00 (5,00-8,25)	Escala de fadiga (Score Global)^b^ 4,00 (3,00-5,56)	0,50 (<0,001)	0,44 (-0,86; 0,70)
Dispneia	7,00 (5,00-8,00)	Escala de dispneia (Score Global)^c^ 3,00 (2,00-4,00)	0,93 (0,007)	0,45 (-0,16; 0,78)
Mal-estar	6,00 (2,00-8,00)	WHOQoL-BREF^d^ 3,14 (2,43-4,14)	-0,59 (0,006)	-0,73 (-2,44; 0,30)

^a^Intervalo de confiança de 95%; ^b^Escala de gravidade de fadiga; ^c^Escala modificada de dispneia do Medical Research Council; ^d^WHOQoL-BREF: domínio I do questionário de qualidade de vida da Organização Mundial da Saúde, versão abreviada.

## Discussão

Este estudo apresentou as etapas de elaboração e avaliação inicial de instrumento, seguindo as recomendações metodológicas da literatura. Elucidou-se que o Formulário de Rastreio e Análise da Trajetória de Recuperação de Sintomas Potencialmente Relacionados à Covid-19 (RRS-COVID-19) possui validade de conteúdo, satisfatória fidedignidade e correlação substancial aos instrumentos contrastados. 

O conjunto de publicações encontradas na revisão de literatura trouxe dificuldades ao mapeamento do construto. Isso porque a revisão de literatura está marcada pela elevada concentração de textos opinativos – algo esperado no início de uma pandemia de doença desconhecida. A seleção cautelosa, à luz da Saúde Baseada em Evidências, levou à construção de ferramenta multicritério, que rastreia e monitora covid-19 aguda ou longa pelo acumulado de 17 manifestações em diferentes estruturas ou sistemas orgânicos, mas em formato desafiador à análise psicométrica.

Neste estudo, reconheceu-se que a taxa de resposta na validação de conteúdo foi limitante, apesar da adoção de cuidadosas práticas, como mensagens e e-mails de lembrete. A baixa adesão dos juízes pôde ser justificada pela condução do trabalho a distância e em período pandêmico (27). A formação farmacêutica, mais frequente entre os juízes, não é, *per si*, problemático. Isso porque o propósito do instrumento foi de rastreio e monitoramento, sem pretensão de realizar diagnóstico médico, analogamente aos testes laboratoriais rápidos amplamente utilizados nas farmácias comunitárias durante a pandemia.

Outra limitação diz respeito à amostra de respondentes, todos convalescentes de covid-19 grave. Como já se reconhece a ocorrência de síndrome da covid-19 pós-aguda em indivíduos com infecção leve ou até assintomáticos (1), observou-se a necessidade de avaliar o desempenho do RRS-COVID-19 para grupos populacionais diferentes.

O valor de α de Cronbach mostrou confiabilidade satisfatória, principalmente por ser instrumento multidimensional, com itens-critério independentes. Embora seja o indicador mais utilizado para escalas múltiplas em saúde, seu uso exclusivo para avaliar a consistência deve ser analisado cautelosamente, dada sua natureza influenciável pelo tamanho e homogeneidade da amostra (28).

A validade concorrente foi analisada pela correlação com ferramentas diversas para itens-critério, diante da ausência de instrumentos padrão-ouro para rastreio de sintomas de covid-19 aguda ou longa. As escalas de fadiga e dispneia foram validadas para brasileiros com doença pulmonar obstrutiva crônica estável (11,12), grupo populacional com características mais próximas as das pessoas com covid-19 longa do que as condições de pacientes oncológicos, público-alvo do instrumento Edmonton Symptom Assessment System (26).

Existem diferentes definições para síndrome da covid-19 pós-aguda que variam principalmente quanto ao marco temporal de início dos sintomas persistentes. Na tentativa de reduzir as discordâncias, a Organização Mundial da Saúde estabeleceu que covid-19 longa é a continuação ou o desenvolvimento de novos sintomas, após 12 semanas da doença aguda, com duração mínima de dois meses (27). A heterogeneidade da apresentação da covid-19 e de suas complicações, associada ao exíguo tempo pós-pandemia para estudar os casos recuperados, é problemática para a elaboração e adequação de instrumentos clínicos. No Brasil, alguns pesquisadores estão atentos a isso, verificando a viabilidade e capacidade discriminatória do instrumento Post-Covid-19 Funtional Scale (29) ou do indicador epidemiológico Disability-Adjusted Life Year, relacionado com a carga global da doença (30). Até o fechamento deste trabalho, não foi encontrado nenhum estudo nacional de avaliação de ferramentas para detecção e monitoramento de sintomas da covid-19 longa. Daí o ineditismo deste estudo.

No contexto da covid-19 longa, há necessidade de se aferir mais do que resultados binários como ter ou não ter determinada condição ou mortalidade. Para esse propósito, estabeleceu-se o consenso de que a abordagem mista com medida dicotômica e escala politômica ordinal seria ideal para aventar toda a gama de manifestações dessa condição. Essas escalas podem ser utilizadas tanto em autorresposta como em entrevista e serve para pesquisa clínica, com obtenção de dados quantitativos com o menor desgaste para o respondente.

Entre os sinais e sintomas inventariados pelo instrumento, observam-se manifestações típicas de morbidade aguda, como febre, mialgias e mal-estar, mas que foram relatados entre 13 e 114 dias após a cura (2,27). É possível que tais problemas persistam devido às sequelas da covid-19 aguda, do seu tratamento e da vulnerabilidade individual decorrente das doenças prévias (3).

O acometimento neural pela infecção viral traz queixas agudas e pode levar a problemas psicossociais, também previstos no instrumento, mediante os itens abrangentes “confusão mental” e “irritabilidade”. A relevância disso consiste no fato de que o adoecimento é um estado de vulnerabilidade às alterações cognitivas e psicológicas que impactam a qualidade de vida. O cenário pandêmico da covid-19 levou a piora do estado de saúde mental de toda sociedade. É necessária a investigação clínico-epidemiológica da carga de saúde mental, para a readequação de políticas e serviços (30).

Esses são os primeiros passos no caminho da criação de instrumento robusto e fidedigno à realidade brasileira, com potencial para ser utilizado na rotina do trabalho em saúde. Iniciativas futuras avaliarão outros parâmetros de confiabilidade e validade desta versão, bem como de suas atualizações e adaptações.

Na perspectiva da urgente necessidade de se caracterizar adequadamente a covid-19, seja aguda ou longa, o instrumento RRS-COVID-19 é inovador ao possibilitar produzir informação comparável, consistente e reprodutível, para subsidiar estratégias no campo da ciência e da assistência em saúde.

## Data Availability

Estão disponíveis, no link, o banco de dados relacionados à revisão da literatura, o quadro de alterações propostas no processo de validação de conteúdo, as demais características dos voluntários da etapa de avaliação da consistência interna e validação concorrente, bem como a versão atual do instrumento: https://drive.google.com/drive/folders/12lTNISpKCF6tQSX-ulw_qD0HINEAfLxp?usp=sharing

## Material Suplementar

Tabela suplementar 1, Tabela suplementar 2, Tabela suplementar 3 e Figura suplementar 1.
